# The blood glucose control levels and influencing factors analysis among diabetes patients in rural areas of Guangxi, China

**DOI:** 10.3389/fendo.2025.1605008

**Published:** 2025-09-05

**Authors:** Jingfeng Chen, Guifen Fu, Xiaoxue Lei, Chaoqun Bai, Lina Wen, Yanping Zhang

**Affiliations:** ^1^ Department of Geriatric Endocrinology and Metabolism, Guangxi Academy of Medical Sciences and the People’s Hospital of Guangxi Zhuang Autonomous Region, Nanning, China; ^2^ Department of Nursing, Guangxi Academy of Medical Sciences and the People’s Hospital of Guangxi Zhuang Autonomous Region, Nanning, China; ^3^ Cardiovascular Medicine Cadre Ward and Geriatric Cardiovascular Medicine Department, Guangxi Academy of Medical Sciences and the People’s Hospital of Guangxi Zhuang Autonomous Region, Nanning, China

**Keywords:** diabetes, glycated hemoglobin, rural areas, glycemic control, associated factors

## Abstract

**Objective:**

Diabetes mellitus is a growing public health concern in China, with the rural areas of Guangxi facing rising prevalence, poor glycemic control, and limited healthcare access despite national efforts to improve diabetes management. This study aimed to evaluate the level of glycated hemoglobin (HbA1c) control and identify associated factors among patients with diabetes in the rural areas of Guangxi, China, to inform strategies for improving diabetes management in these regions.

**Methods:**

A multistage stratified random sampling method was employed. In the first stage, five cities (Nanning, Guilin, Hechi, Chongzuo, and Yulin) were randomly selected, each representing a geographical region of Guangxi (central, eastern, southern, western, and northern). In the second stage, three counties were randomly selected from each city, yielding a total of 15 counties. One county-level hospital with a general internal medicine department was selected in each county for patient recruitment. Data on demographic characteristics (e.g., sex, age, household income, type of medical insurance, educational level, and disease duration) were collected, and laboratory testing was conducted to measure HbA1c levels. Glycemic control was defined as an HbA1c level< 7%. Multivariate logistic regression was used to identify factors associated with glycemic control.

**Results:**

A total of 2,178 patients with diabetes were included, of whom 1,204 (55.28%) were men and 974 (44.72%) were women. The mean age was 63.25 ± 12.71 years, and the mean duration of diabetes was 7.96 ± 4.07 years. The overall HbA1c control rate was 22.68%. Logistic regression analysis revealed that older age (OR = 1.026, 95% CI: 1.017–1.036), longer disease duration (OR = 1.137, 95% CI: 1.104–1.171), use of oral hypoglycemic agents (OR = 0.485, 95% CI: 0.377–0.624), insulin therapy (OR = 0.425, 95% CI: 0.388–0.534), and higher educational level (e.g., primary school: (OR = 6.507, 95% CI: 3.076–13.767); junior high school: (OR = 5.557, 95% CI: 2.818–10.955); senior high school: (OR = 2.848, 95% CI: 1.485–5.462); college: (OR = 2.479, 95% CI: 1.285–4.782); and bachelor’s degree: (OR = 1.915, 95% CI: 0.943–3.889), and higher annual per capita household income (OR = 0.626, 95% CI: 0.528–0.830) were significantly associated with glycemic control (*p*< 0.05).

**Conclusion:**

The HbA1c control rate among patients with diabetes in rural Guangxi was relatively low (22.68%). Targeted interventions should focus on patients who are older, have a longer disease duration, are not receiving antidiabetic treatment, have lower educational levels, or have lower income levels to improve glycemic management in rural areas.

## 1 Introduction

Diabetes mellitus is a chronic metabolic disorder characterized by persistent hyperglycemia (1). It has become a major global public health concern, with its prevalence steadily increasing because of population aging, improved living conditions, and increased life expectancy (2). According to the 10th edition of the *IDF Diabetes Atlas* (3), an estimated 537 million individuals worldwide had diabetes in 2021. This number is projected to reach 643 million by 2030 and 783 million by 2045. Additionally, approximately 541 million people were estimated to have impaired glucose tolerance in 2021.

Currently, China has the largest population of individuals with diabetes, and its prevalence is increasing annually. In 2021, approximately 141 million people in China were living with diabetes (3), and this number is projected to exceed 174 million by 2050 (4). As the disease progresses, persistent hyperglycemia can lead to various complications. Chronically elevated blood glucose levels are associated with both macrovascular (such as coronary heart disease and stroke) and microvascular complications (including diabetic nephropathy and retinopathy) (5). Glycemic control is essential for reducing morbidity and mortality associated with type 2 diabetes mellitus. Clinical trials have shown that maintaining HbA1c levels below 7% significantly lowers the risk of diabetes-related complications, including neuropathy, retinopathy, and nephropathy. Additionally, a 1.0% reduction in HbA1c is associated with a decrease in the risk of diabetes-related mortality by 21%, myocardial infarction by 14%, amputation or death from peripheral vascular disease by 43%, and microvascular complications by 37% (6). Therefore, glycemic control remains a fundamental target in the treatment and self-management of diabetes (7).

Furthermore, diabetes imposes a substantial economic burden on both individuals and society. In 2019, approximately 10% of the global healthcare expenditure—an estimated $760 billion—was attributable to diabetes. China ranks second globally in diabetes-related healthcare costs, with expenditures reaching $109 billion in 2021 (8). In response to the growing burden, China incorporated diabetes management into its basic public health services at the primary care level in 2009. However, nationwide surveys have shown that among patients aged over 35 years, glycemic control and standardized diabetes management remain inadequate, particularly in rural populations (9).

The Guangxi Zhuang Autonomous Region, located in southern China, is an economically underdeveloped area where rural residents often have lower educational attainment and limited health literacy. The prevalence of diabetes is rapidly increasing in this population (10). To better understand glycemic control in this context, we conducted a cross-sectional study of adult residents with diabetes in rural Guangxi. This study aimed to assess HbA1c control levels and identify associated factors to inform targeted interventions for improving diabetes care in rural settings.

## 2 Materials and methods

### 2.1 Study participants

Between January and July 2022, a multistage stratified sampling method was employed to recruit participants. In the first stage, five cities—Nanning, Guilin, Hechi, Chongzuo, and Yulin—were randomly selected to represent the five geographic regions of the Guangxi Zhuang Autonomous Region (central, eastern, southern, western, and northern). In the second stage, three counties were randomly selected from each city, resulting in 15 counties selected for this study. One hospital (specifically, the internal medicine department) in each county was selected, and 152 patients with diabetes were surveyed per hospital. A total of 2,280 patients were initially enrolled. Inclusion criteria were as follows: (1) diagnosis of diabetes based on the 2020 criteria of the Chinese Diabetes Society (11), (2) age ≥ 18 years, and (3) registered household and current residence within the selected survey area. Exclusion criteria included the following: (1) patients with gestational diabetes; (2) patients with dementia or other psychiatric disorders, as identified through medical records or reported by family members; and (3) patients in the acute phase of illness and unable to cooperate with the investigation. All participating hospital administrators provided institutional consent, and all individual participants provided written informed consent.

### 2.2 Methods

#### 2.2.1 Questionnaire survey

A structured questionnaire developed by the research team was used to collect data on the demographic and clinical characteristics. Demographic variables included age, sex, marital status, educational level, and annual per capita household income (9), while behavioral variables included smoking and alcohol consumption. Clinical variables included duration of diabetes, HbA1c levels, use of oral antidiabetic drugs or insulin, presence of diabetes-related complications, and family history of diabetes.

#### 2.2.2 Laboratory testing

On the second day of hospitalization, venous blood samples (2 ml) were collected from each participant (after > 10h of fasting) for HbA1c testing. Blood samples were collected by hospital medical staff, and HbA1c levels were measured using high-performance liquid chromatography in each hospital’s laboratory.

### 2.3 Operational definitions

Glycemic control was defined according to the *Chinese Guidelines for the Prevention and Treatment of Type 2 Diabetes Mellitus (2020 edition)* (7) as HbA1c<7.0%. Glycemic control rate was calculated as follows:


$$\begin{array}{c} control\;rate\;\left(\%\right)=(number\;of\;patients\;with\;HbA1c\;\\ <7.0\%/total\;number\;of\;patients\;surveyed)\times 100\% \end{array}$$

Diabetes-related complications included diabetic nephropathy, diabetic foot, lower limb vascular disease, retinopathy, and neuropathy—all confirmed through clinical diagnosis. Smoking was defined as continuous or cumulative smoking for ≥6 months. Categories were defined as follows: regular smoking (≥1 cigarette/day), occasional smoking (>4 times/week but<1 cigarette/day), and former smoker (quit smoking for >6 months). Alcohol consumption was categorized as regular drinking (more than once per week but not daily), occasional drinking (once per week or less), and former drinker (quit for more than 6 months). For analysis, smoking and drinking were classified as “yes” (currently regular use) or “no” (never, former, or occasional use).

### 2.4 Ethical considerations

The study protocol was approved by the Ethics Committee of the corresponding hospital (approval number: KT-KJT-2021-26). The purpose, voluntary nature, and confidentiality of the study were explained using a questionnaire. All hospital administrators and participants provided informed consent.

### 2.5 Data collection

Data were collected through in-person questionnaire-based interviews and blood sampling conducted at local hospitals. Investigators provided standardized instructions to guide participants in completing the questionnaires. General information and HbA1c test results from all 15 hospitals were recorded and entered into Excel spreadsheets, which were electronically submitted to the research team. All data were independently verified by two researchers and subsequently imported into SPSS for analysis. Data were stored on password-protected computers to ensure confidentiality.

### 2.6 Statistical analysis

A database was created using Microsoft Excel, and statistical analyses were performed using SPSS version 25.0. Continuous variables with normal distribution were presented as mean ± standard deviation, and group comparisons were conducted using t-tests. Categorical variables were described as frequencies and compared using the chi-square (χ²) test. Factors associated with HbA1c control were analyzed using multivariate logistic regression. Statistical significance was set at *p*< 0.05.

## 3 Results

### 3.1 Baseline characteristics of participants

A total of 2,280 rural patients with diabetes were surveyed, of whom 2,178 provided valid responses and were included in the final analysis. The participants had a mean age of 63.25 ± 12.71 years and a mean duration of diabetes of 7.96 ± 4.07 years. Among these participants, 55.28% were men, indicating a higher proportion of men than women. Regarding socioeconomic characteristics, 31.43% had completed junior high school, while 37.74% had an annual per capita household income of ≥18,931 RMB. A family history of diabetes was reported by 14.55% of the respondents. For glycemic control, 22.68% of participants had an HbA1c level<7.0%, while 77.32% had an HbA1c level ≥7.0%. Detailed demographic and clinical characteristics are presented in **Table 1**.

**Table 1:** General information of surveyed rural diabetic patients (n=2178).

Variable	Group	Count (Percentage %)
Sex	Male	1204 (55.28)
	Female	974 (44.72)
Marital Status	Married	2020 (92.75)
	Unmarried or Widowed	158 (7.25)
Educational Level	No formal education	256 (11.75)
	Primary School	504 (23.14)
	Junior High School	684 (31.41)
	High School	507 (23.28)
	Associate Degree	180 (8.26)
	Bachelor’s Degree	47 (2.16)
Average Family Disposable Income	< 18931 Yuan	1356 (62.26)
	≥ 18931 Yuan	822 (37.74)
Family History of Diabetes	Yes	317 (14.55)
	No	1861 (85.45)
HbA1c Control Status	< 7.0	494 (22.68)
	≥ 7.0	1684 (77.32)

### 3.2 HbA1c control status

Among the 2,178 rural patients with diabetes, 494 had HbA1c levels<7.0%, yielding a glycemic control rate of 22.68%. The remaining 1,684 patients (77.32%) had HbA1c levels ≥7.0%. Patients with uncontrolled HbA1c levels were generally older and had a longer duration of diabetes than those with controlled HbA1c levels. Characteristics associated with poorer HbA1c control included being male, having no formal education, being unmarried or widowed, having a lower household income, smoking, alcohol consumption, the presence of diabetes-related complications, the absence of a family history of diabetes, and not receiving oral hypoglycemic agents or insulin therapy (all *p<* 0.05). Detailed results are presented in **Table 2**.

**Table 2:** Comparison of blood sugar control rate among rural diabetic patients.

Variable	Survey Cases	HbA1c Controlled (n=494)	HbA1c Uncontrolled (n=1684)	Control Rate (%)	Statistics	p-value
Age (years)	2178	62.03 ± 12.593	63.61 ± 12.727	–	2.442^a^	0.015
Sex					13.035^b^	<0.001
Male	1204	238	966	19.77		
Female	974	256	718	26.28		
Educational Level					88.891^b^	<0.001
Illiterate	256	28	228	10.94		
Primary School	505	70	434	13.86		
Junior High School	684	167	517	24.42		
High School	507	142	365	28.01		
Associate Degree	180	63	117	35.00		
Bachelor’s Degree	47	24	23	51.06		
Marital Status					3.039^b^	0.081
Married	2020	467	1553	23.12		
Unmarried or Widowed	155	27	131	17.42		
Average Family Disposable Income					60.290^b^	<0.001
<18931 Yuan	1356	234	1122	17.26		
≥18931Yuan	822	260	562	31.63		
Smoking					0.257^b^	0.612
Yes	396	86	310	21.71		
No	1782	408	1374	22.89		
Alcohol Consumption					3.576^b^	0.059
Yes	496	97	399	19.56		
No	1682	397	1285	23.60		
Duration of Diabetes	2178	6.44 ± 3.73	8.40 ± 4.06	–	9.636^a^	<0.001
Complications					4.559^b^	0.033
Yes	1571	342	1229	21.77		
No	607	152	455	25.04		
Family History of Diabetes					12.227^b^	<0.001
Yes	317	96	221	30.28		
No	1861	398	1463	21.38		
Oral Hypoglycemic Agents					67.615^b^	<0.001
Yes	1349	384	965	28.47		
No	829	110	719	13.27		
Insulin Treatment					139.552^b^	<0.001
Yes	913	321	592	35.16		
No	1265	173	1092	13.68		

^a^, t-test; ^b^, χ^2^ test.

#### 3.2.1 Comparison of HbA1c control across sociodemographic characteristics

A comparison of sociodemographic and clinical characteristics between the HbA1c-controlled and uncontrolled groups is presented in **Table 2**. Significant differences were observed between the two groups in age (*t* = 2.442, *p*< 0.015), sex (χ² = 13.035, *p*< 0.001), family history of diabetes (χ² = 12.227, *p*< 0.001), use of oral antidiabetic drugs (χ² = 67.615, *p*< 0.001), use of insulin therapy (χ² = 139.552, *p*< 0.001), educational level (χ² = 88.891, *p<* 0.001), annual per capita household income (χ² = 60.290, *p<* 0.001), duration of diabetes (*t* = 9.636, *p*< 0.015), and presence of diabetes-related complications (χ² = 4.559, *p*< 0.001).

### 3.3 Multivariate logistic regression analysis of factors associated with HbA1c control

Multivariate logistic regression analysis was performed to identify factors independently associated with HbA1c control, with glycemic control (HbA1c<7.0%) as the dependent variable (0 = controlled, 1 = uncontrolled). Independent variables included age, sex, family history of diabetes, use of oral antidiabetic drugs, insulin therapy, educational level, per capita annual household income, duration of diabetes, and presence of complications. The analysis revealed that age, use of oral antidiabetic drugs, insulin therapy, educational level, income, and duration of diabetes were significantly associated with HbA1c control (*p*< 0.05) (**Table 3**). Men had a slightly higher likelihood of achieving glycemic control than did women (OR: 0.800; 95% CI: 0.639–1.002). Each additional year of age was associated with a 2.6% increase in the risk of poor glycemic control (OR: 1.026; 95% CI: 1.017–1.036). Participants with a family history of diabetes were more likely to have poor glycemic control than those without (OR: 1.311; 95% CI: 0.973–1.766), although the difference was not significant. Regarding treatment, both the use of oral antidiabetic drugs (OR: 0.485; 95% CI: 0.377–0.624) and insulin therapy (OR: 0.425; 95% CI: 0.338–0.534) were significantly associated with better glycemic control. Higher educational attainment was associated with improved HbA1c control. Compared with participants who have no formal education, those with primary education (OR: 6.507; 95% CI: 3.076–13.767), junior high school education (OR: 5.557; 95% CI: 2.818–10.955), senior high school education (OR: 2.848; 95% CI: 1.485–5.462), vocational college education (OR: 2.479; 95% CI: 1.285–4.782), and bachelor’s degrees or above (OR: 1.915; 95% CI: 0.943–3.889) had more favorable control rates. Economically, participants with a per capita annual household income ≥18,931 RMB were significantly more likely to achieve HbA1c control than those with lower income levels (OR: 0.662; 95% CI: 0.528–0.830). Additionally, each 1-year increase in diabetes duration was associated with a 13.7% increase in the risk of poor glycemic control (OR: 1.137; 95% CI: 1.104–1.171). No significant association was found between the presence of diabetes-related complications and HbA1c control (OR: 0.945; 95% CI: 0.739–1.209; *p* = 0.652).

**Table 3:** Logistic regression analysis of HbA1c achievement rate in rural diabetic patients.

Variable	β	SE	*Waldχ^2^*	*p*	OR (95%CI)
Constant	-3.874	0.499	60.217	<0.001	–
Age (years)	0.026	0.005	31.083	<0.001	1.026 (1.017,1.036)
Sex					
Male	-0.223	0.115	3.780	0.052	0.800 (0.639,1.002)
Female	–	–	–	–	–
Family History of Diabetes					
Yes	0.271	0.152	3.173	0.075	1.311 (0.973,1.766)
No	–	–	–	–	–
Oral Hypoglycemic Agents					
Yes	-0.724	0.129	31.432	<0.001	0.485 (0.377,0.624)
No	–	–	–	–	–
Insulin Treatment					
Yes	-0.855	0.117	53.482	<0.001	0.425 (0.388,0.534)
No	–	–	–	–	–
Educational Level			54.784	<0.001	
Illiterate	–	–	–	–	–
Primary School	1.873	0.382	23.997	<0.001	6.507 (3.076,13.767)
Junior High School	1.715	0.346	24.521	<0.001	5.557 (2.818,10.955)
High School	1.047	0.332	9.925	0.002	2.848 (1.485,5.462)
Associate Degree	0.908	0.335	7.333	0.007	2.479 (1.285,4.782)
Bachelor’s Degree	0.650	0.361	3.234	0.072	1.915 (0.943,3.889)
Average Family Disposable Income					
<18931 Yuan	–	–	–	–	–
≥18931Yuan	-0.412	0.115	12.828	<0.001	0.626 (0.528,0.830)
Duration of Diabetes	0.129	0.015	73.340	<0.001	1.137 (1.104,1.171)
Complications					
Yes	-0.057	0.125	0.203	0.652	0.945 (0.739,1.209)
No	–	–	–	–	–

## 4 Discussion

Diabetes is a prevalent chronic disease, and maintaining stable blood glucose levels is critical for preventing and managing its complications. However, glycemic control varies across regions owing to differences in geographical location, socioeconomic status, dietary habits, and healthcare accessibility. This study aimed to assess the status of glycemic control and its influencing factors among rural residents with diabetes in the Guangxi Zhuang Autonomous Region, China.

Our findings revealed that the HbA1c control rate (defined as HbA1c<7.0%) among rural patients in Guangxi was 22.68%, with the majority (77.32%) exhibiting poor glycemic control (HbA1c ≥7.0%). This control rate was significantly lower than those reported in Zhejiang Province (47.89%) (12) and Shanghai (39.0%) (13), indicating that the glycemic control situation in rural Guangxi is particularly concerning.

Although diabetes management in Guangxi follows the national basic public health service standards, significant disparities in glycemic control remain. These differences may be attributed to regional variations in economic development, healthcare resources, and residents’ health literacy. Therefore, targeted measures are necessary to enhance blood glucose control among rural populations in the region.

Multivariate logistic regression analysis identified older age as a significant factor associated with poor HbA1c control. This may be attributed to age-related declines in pancreatic β-cell function and impaired glucose metabolism, making it more difficult to maintain glycemic control (14).

Longer diabetes duration was also associated with poor glycemic control. As the disease progresses, pancreatic β-cell function deteriorates, and treatment effectiveness may decline, increasing the likelihood of uncontrolled blood glucose levels (15). Household income also emerged as an important factor, as patients with higher annual per capita income were more likely to achieve HbA1c targets. This finding aligns with that of a study by Ibrahim et al. (16), which showed that low-income patients often have poorer glycemic control. Financial constraints may limit access to quality healthcare, affordable medications, and adequate nutrition. Diabetes is a resource-intensive disease that requires consistent investment in monitoring and treatment. Consequently, rural patients with limited income may be unable to afford glucometers, test strips, or needles, resulting in less frequent blood glucose monitoring and suboptimal management. In contrast, higher income patients are more likely to engage in regular monitoring and proactive disease management. Additionally, health insurance has been shown to ease the financial burden on patients and improve glycemic control (17). Therefore, policy interventions—such as providing free or subsidized glucose-monitoring supplies and enhancing healthcare services in rural areas—are crucial to alleviate the economic barriers to effective diabetes management.

Educational level was found to be another key determinant of glycemic control. In this study, most patients had a junior high school education (31.41%). Patients with higher educational attainment generally had better glycemic control, consistent with the findings of Sonmez et al. (18) and Nigussie et al. (19). Education is strongly associated with health literacy, as patients with higher educational levels tend to have a better understanding of disease management and are more proactive in seeking medical care. For those with limited literacy, health education strategies should be tailored using simple, accessible language and delivered more frequently to enhance their understanding of self-management and improve treatment adherence.

Furthermore, regular use of oral antidiabetic drugs or insulin significantly improves glycemic control (19). Our findings support this association, as patients receiving oral medications or insulin therapy were more likely to achieve target HbA1c levels. Oral antidiabetic medications act through various mechanisms, such as enhancing insulin secretion, improving insulin sensitivity, or regulating hepatic glucose production (20). However, some oral agents can cause side effects such as hypoglycemia, dizziness, and nausea (21). Therefore, patients must use these medications under medical supervision to prevent inappropriate dosing and adverse outcomes. Insulin, a protein hormone secreted by pancreatic β cells, promotes glucose uptake and storage as glycogen or fat, thereby lowering blood glucose levels. It is commonly used to treat diabetes (22). Patients initiating insulin therapy should follow clinical guidelines and select appropriate insulin formulations based on their glycemic profiles (23). Common adverse effects of insulin include hypoglycemia and weight gain. Therefore, severe symptoms should be promptly addressed, and the dosage adjusted by a physician when necessary.

### 4.1 Limitations

This study had a few limitations. First, it was a multicenter cross-sectional survey conducted in the internal medicine departments of 15 county-level hospitals in the Guangxi Zhuang Autonomous Region. All participants were patients who actively sought medical care, excluding undiagnosed or untreated individuals with diabetes in rural areas. This introduces a selection bias, as those who visit hospitals may exhibit greater health literacy and better treatment adherence. Therefore, the reported HbA1c control rate may have been overestimated and should not be directly generalized to the broader rural diabetic population in Guangxi.

Second, the sampling process involved randomly selecting one prefecture-level city from each of the five geographical regions of Guangxi (central, eastern, southern, western, and northern), followed by the random selection of three counties within each city. Although this approach aimed to account for geographic and healthcare diversity, it did not incorporate stratified weighting based on rural population proportions. Consequently, areas with larger rural populations were not given proportionally greater sampling weights. Thus, the sample may not fully represent all rural patients with diabetes in the region, and the findings primarily reflect those who actively seek medical care.

Third, because of practical constraints, the types of HbA1c testing equipment used varied across participating hospitals. Although all sites utilized high-performance liquid chromatography, and testing was conducted by trained laboratory professionals, minor measurement variability between instruments may persist. To minimize potential bias, we adopted an HbA1c threshold of<7.0%, as recommended by the *Chinese Guidelines for the Prevention and Treatment of Type 2 Diabetes Mellitus (2020 edition)*. Nevertheless, caution is advised when interpreting the HbA1c control rate data.

### 4.2 Conclusion

The HbA1c control rate among rural patients with diabetes in the Guangxi Zhuang Autonomous Region remains suboptimal. Targeted attention should be directed toward individuals who are older, have a longer duration of diabetes, are receiving oral hypoglycemic or insulin therapy, have lower educational levels, or belong to low-income households. Tailored lifestyle interventions, along with enhanced training of community healthcare providers, are essential. Improving patients’ health literacy and reinforcing their awareness of and responsibility for diabetes self-management may enhance adherence to follow-up care and lead to better outcomes in community-based diabetes control programs.

## Data Availability

The original contributions presented in the study are included in the article/supplementary material. Further inquiries can be directed to the corresponding author.
